# Proteomic Analysis of the Rat Canalicular Membrane Reveals Expression of a Complex System of P4-ATPases in Liver

**DOI:** 10.1371/journal.pone.0158033

**Published:** 2016-06-27

**Authors:** Pururawa Mayank Chaubey, Lia Hofstetter, Bernd Roschitzki, Bruno Stieger

**Affiliations:** 1 Department of Clinical Pharmacology and Toxicology, University Hospital Zürich, Zürich, Switzerland; 2 Functional Genomics Center Zürich, University of Zürich/ETH Zürich, Zürich, Switzerland; RWTH Aachen, GERMANY

## Abstract

Transport processes in the canalicular membrane are key elements in bile formation and are the driving force of the enterohepatic circulation of bile salts. The canalicular membrane is constantly exposed to the detergent action of bile salts. One potential element protecting the canalicular membrane from the high canalicular bile salt concentrations may be bile salt resistant microdomains, however additional factors are likely to play a role. To obtain more insights into the molecular composition of the canalicular membrane, the proteome of highly purified rat canalicular membrane vesicles was determined. Isolated rat canalicular membrane vesicles were stripped from adhering proteins, deglycosylated and protease digested before subjecting the samples to shot gun proteomic analysis. The expression of individual candidates was studied by PCR, Western blotting and immunohistochemistry. A total of 2449 proteins were identified, of which 1282 were predicted to be membrane proteins. About 50% of the proteins identified here were absent from previously published liver proteomes. In addition to ATP8B1, four more P4-ATPases were identified. ATP8A1 and ATP9A showed expression specific to the canalicular membrane, ATP11C at the bLPM and ATP11A in an intracellular vesicular compartment partially colocalizing with RAB7A and EEA1 as markers of the endosomal compartment. This study helped to identify additional P4-ATPases from rat liver particularly in the canalicular membrane, previously not known to be expressed in liver. These P4-ATPases might be contributing for maintaining transmembrane lipid homeostasis in hepatocytes.

## Introduction

Bile formation involves the hepatocellular uptake of cholephilic substances, e.g. bile salts from the sinusoidal blood plasma, the diffusion of these substances across the hepatocyte and their export across the apical (canalicular) membrane into bile [1]. Bile is delivered from the liver to the intestine, where the bile salts help to assist the digestion of fat and facilitate the absorption of lipids. In the small intestine the majority of the bile salts are reabsorbed and transported back by the portal circulation to the liver for uptake into hepatocytes, followed by a new secretion into bile, i.e. they undergo enterohepatic circulation. In addition to containing bile salts, bile is also rich in phospholipids (almost exclusively phosphatidylcholine (PC)) and cholesterol. Bile salts together with PC form mixed micelles. These mixed micelles protect the biliary tree from the toxic action of bile salts [2] and act as acceptors for poorly water soluble substances like cholesterol, the excretion of which into bile at the canalicular membrane is facilitated by ABCG5/ABCG8. A PC translocator, namely MDR3 [3] regularly replenishes PC in the outer leaflet of the canalicular membrane (cLPM). The bile salt export pump (BSEP) transports bile salts across the cLPM and constitutes the rate limiting step of hepatocellular bile salt transport. Consequently BSEP acts as a key player in hepatocellular bile salt transport and is driving the enterohepatic circulation of bile salts [1]. Current information suggests that a complex interplay of BSEP, MDR3 and ABCG5/ABCG8 is crucial for biliary lipid secretion. In addition, microdomains present in the cLPM may protect this side of the hepatocyte from the detergent action of the high bile salt concentration present in the canaliculus [4, 5]. Mutations in the genes coding for BSEP, MDR3 or ATP8B1 lead in humans to progressive familial intrahepatic cholestasis. ATP8B1 is an aminophospholipid flippase involved in maintaining the lipid asymmetry and the proper cholesterol content of the cLPM [6]. Despite a lot of knowledge on the pathophysiologic consequences of mutations in these three genes, wide variations in the clinical phenotypes resulting from such mutations are observed and are only partially explained [7]. In addition, in some patients with progressive familial intrahepatic cholestasis, no mutations in the known disease-causing genes are found [8]. To expand our current knowledge on the protein composition of the cLPM we determined the proteome of highly purified rat cLPM by using a shot gun proteomics approach.

## Experimental Procedures

### Chemicals and reagents

All chemicals used were purchased from Sigma Aldrich (Switzerland) unless otherwise indicated. All the primers (S1 Table) for real time PCR were purchased from Life Technology (Switzerland). The polyclonal antibodies against ATP8A1 was obtained from Proteintech (cat# 21565-1-AP, Rosemont, IL, USA). The polyclonal antibody against ATP11C (cat# ab175055) and the monoclonal antibody against RAB7 (cat# ab126712) were ordered from Abcam (Cambridge, MA, USA). The polyclonal antibody against ATP11A (cat# HPA035583) and the monoclonal antibody against ATP9A (cat# WH0010079M2) were obtained from Sigma (St. Louis, MO, USA). The monoclonal antibody against EEA1 is produced by BD Biosciences (cat# 610457, Franklin Lakes, NJ, USA). The HRP-conjugated goat-anti-rabbit antibody was purchased from GE Healthcare (cat# RPN4301, Little Chalfont, UK) and the HRP-conjugated goat anti mouse antibody from Pierce (cat# NA931V, Waltham, MA, USA). The Alexa fluor488 labeled goat-anti-rabbit antibody was from ThermoFisher Scientific (cat# A11008, Waltham, MA, USA) and the Cy3-conjugated affiniPure goat-anti-mouse antibody from Jackson ImmunoResearch (cat# 115-165-146, West Grove, PA, USA).

### Animals

Male Sprague–Dawley (SD) rats (180–200 g) obtained from Harlan (Horst, The Netherlands) received humane care in accordance with local and federal regulations and were kept under standard conditions at the animal facility of the University Hospital. The rats were sacrificed by decapitation and the animal experiments were approved by the "Veterinäramt des Kantons Zürich".

### Sub-cellular fractions

#### Rat liver microsomes

Fresh liver was homogenized in homogenization buffer (250mM sucrose, 10mM Tris/HCL pH 7.6, freshly added 1μg/ml antipain/leupeptin, 5 μg/ml aprotinin, 40 μg/ml PMSF, 50 μg/ml benzamidine, 0.5 μg/ml pepstatin A) using a Potter Elvehjem homogenizer (Kontes Glass, Vineland, NJ, USA) on ice. The homogenate was processed as described in [9] and the resulting microsomes were suspended in 250 mM sucrose and stored in liquid nitrogen until use.

#### Rat liver mitochondria

Ice cold preparation medium (210 mM mannitol, 70 mM Sucrose, 0.5 mM EDTA, 10 mM Tris/HCL pH 7.4) was added to finely chopped livers, which had been quickly removed from the sacrificed animal. The liquid was decanted and washing with preparation medium was repeated twice. The tissue was homogenized in a Potter Elvehjem homogenizer (Kontes Glass, Vineland, NJ, USA) as a 10% (w/v) tissue suspension by 3–4 strokes. The homogenate was centrifuged at 800 g_av_ for 10 min. This supernatant was centrifuged at 10,000 g_av_ for 8 min. The pellet was carefully resuspended in preparation medium and sedimented again. The final pellet was suspended in about 2 ml preparation medium and stored frozen until use.

#### Rat liver canalicular plasma membrane (cLPM) and basolateral liver plasma membrane (bLPM)

Rat cLPM and bLPM vesicles were isolated by a standard procedure as described in detail in [10, 11] and stored in liquid nitrogen until use.

### Mass-spectrometric analysis of cLPM

2 mg of cLPM were re-suspended and homogenized in 0.2 M Na_2_CO_3_ along with protease inhibitors (Mini Complete, Roche Diagnostics, Rotkreuz, Switzerland) and were centrifuged at 100,000 g_av_ for 1 h at 4°C. The protein estimation was carried out using the BCA protein assay kit (Interchim, Monluçcon, France).

#### In solution digest

2 mg of the extracted cLPM from above were first subjected to PNGase F (New England Biolabs, Ipswich, MA, USA) treatment overnight at 37°C as instructed by the supplier on a thermoshaker (500 rpm) for deglycosylation of the glycoproteins. This was followed by diluting the proteins in 20 mM ammonium bicarbonate (pH 8) and 0.1% (w/v) Rapigest SF surfactant (Waters, Elstree, UK). The samples were reduced using 5 mM dithiothreitol for 30 min at 60°C and alkylated using 45 mM iodoacetamide for 30 min in the dark at room temperature (RT). The reaction was quenched by adding 30 mM dithiothreitol at RT for 10 min. The resulting sample was then digested using trypsin (sequencing grade; Promega, Dübendorf, Switzerland) at a ratio of 1:20 at 37°C for 3.5 h at 700 rpm on a thermoshaker followed by centrifugation for 10 min at maximum speed in a table top centrifuge. The supernatant (S1) was collected and kept aside. The pellet was further digested by adding chymotrypsin and trypsin at a ratio of 1:100 each overnight at 37⁰C at 700 rpm. The reaction was stopped by adding 50% (v/v) acetonitrile and 0.1% (v/v) trifluroacetic acid at 37°C, 30 min rotating at 700 rpm. After centrifugation for 10 min, the supernatant (S2) was collected and pooled with S1 for further analysis.

#### Pre-fraction by strong cation exchange (SCX) chromatography

To decrease the complexity of peptides, pre-fractionation was performed using SCX chromatography. Peptides were desalted using a C18 column, vacuum dried and resuspended in buffer A (10 mm KH_2_PO_4_, pH 2.7, 30% (v/v) acetonitrile), before loading onto the polysulfoethyl aspartamide A column (2.1 × 200 mm; PolyLC, Columbia, MD, USA) on an Agilent HP1100 binary HPLC system (Agilent Technologies, Santa Clara, CA, USA). Peptides were eluted using an increasing KCl gradient (10–40 min, 0–30% buffer B; 40–60 min 30–100% buffer B; (buffer B: 10 mm KH_2_PO_4_, pH 2.7, 500 mm KCl 30% (v/v) acetonitrile) and fractions of ~0.6 ml were collected. Eluted peptides were pooled based on the chromatogram into seventeen fractions. The fractions were desalted using ZipTips (Merck Millipore, Darmstadt, Germany) following protocol of the manufacturer.

#### MS acquisition

The desalted peptides were vacuum-concentrated and resuspended in 3% (v/v) acetonitrile and 0.1% (v/v) formic acid. The MS analysis was performed with a LTQ Orbitrap XL mass spectrometer (Thermo Fisher Scientific, Waltham, MA, USA) and one replicate of Rep_3 was analyzed with a LTQ-FT-ICR Ultra mass spectrometer (Thermo Fisher Scientific, Waltham, MA, USA), both instruments equipped with an Eksigent nano LC system (Eksigent Technologies, Dublin, CA, USA). The peptides were separated by HPLC by using a homemade C18 reversed phase tip-column (Magic C18 AQ, 3 μM, 200 Å, Bischoff GmbH, Leonberg, Germany) [12] with a flow rate of 200 nl/min using solvent A (0.1% (v/) formic acid in H_2_O) and solvent B (0.1% (v/v) formic acid in acetonitrile) and the following gradient: 3% B to 50% B in 50 min, 50% B to 58% B in 8 min and 58% B to 97% B in 2 min. 97% B was kept for 7 min to wash the column and afterwards the column was re-equilibrated with 3% B for 9 min. High accuracy mass spectra were acquired in a mass range of 300–2000 m/z with LTQ-Orbitrap and LTQFT-ICR Ultra, respectively. Up to 6 data dependent MS/MS were recorded in parallel in the linear ion trap of the most intense MS signals with charge state 2+ or 3+ using collision-induced dissociation. Target ions already selected for MS/MS were dynamically excluded for 60 s.

#### Data refinement and protein identification

Mascot distiller 2.3 was used to extract the peak list from all MS raw files [13]. The peak lists were searched against Rat UniProt database (http://www.uniprot.org/ release 2013, 28781 entries) that has been concatenated with its reversed sequences and elongated by common contaminants (e.g. human keratins and bovine proteins) using Mascot 2.5 (Matrix Science, London, UK, www.matrixscience.com) [14]. The carbamidomethyl (Cys) was kept as fixed modification and the oxidation (Met), deamidation (Asn), acetylation N-term protein and Gln → pyro-Glu (N-term Gln) were set as variable modification. Precursor ions subjected to MS/MS were searched with a parent mass error tolerance of 10 ppm and a fragment ion error tolerance of 0.8 Dalton and a maximum of one missed cleavage on tryptic peptides were allowed. Mascot results were uploaded to Scaffold 4.5 (Proteome Software, Portland, OR, USA) [15] and thresholds for peptide and protein identification were set to a false discovery rate of 1%. Further data analysis was done by using Microsoft Excel 2010 (Microsoft Corp, Redmond, WA, USA) and Pivot tables. The proteomics data have been deposited in the PRIDE/ProteomeXchange consortium [16] with the data set identifier PXD003299.

### Semi-quantitative real-time polymerase chain reaction

Total mRNAs were isolated from rat livers using a protocol previously described by Eloranta *et al*. [17]. Double stranded complementary DNAs (cDNAs) of rat P4-ATPases (*Atp8a1*, *Atp8b1*, *Atp9a*, *Atp11a1*, *Atp11c*), and a P5-ATPase (*Atp13a1*) were amplified from total mRNA using TaqMan master mix and primers (Life Technologies, Portland, CA, USA) (S1 Table) by reverse transcription—polymerase chain reaction (qRT-PCR). Transcript levels were measured using an ABI Prism 7900HT fast qRT-PCR system (Applied Biosystems, Carlsbad, CA, USA). Measured transcript values were normalized against the rat β-actin and GAPDH cDNA levels using the comparative threshold cycle method.

### Western Blotting

Proteins were identified in liver subcellular fractions using Western blotting as described in [18]. The following antibody dilutions were used: ATP8A1 at 1:500, ATP9A at 1:250, ATP11A at 1:250 and ATP11C at 1:250. The different primary antibodies were detected using horse-radish peroxidase (HRP)-conjugated goat-anti-rabbit or HRP-conjugated goat-anti-mouse antibodies. The bands were visualized by using the ECL chemiluminescence kit (Interchim, Montluçon, France).

### Protein Immunostaining

#### Liver perfusion and immunohistochemistry (IHC)

Rat livers were perfused *in situ* with 4% (w/v) paraformaldehyde in 250 mM sucrose for 10 min. Livers were cut into 0.5 cm cubes and were stored in 0.02% (w/v) sodium azide and 100 mM sodium phosphate buffer pH 7.4 at 4°C until further use. The perfused and non-perfused liver tissues were equilibrated in 30% (w/v) sucrose solution overnight and then embedded in Tissue-Tek^*®*^ O.C.T. *™ (*Sakura Finetek, Torrance, CA, USA) before performing the cryosectioning. Frozen 5*μm* sections of perfused and non-perfused livers were cut using a cryostat (Leica CM 3050 S, Leica, Wetzlar, Germany)), dried at RT for 2–3 h and later stored at 4°C until further use. Immunostaining for various P4-ATPases was performed using the same antibodies as used for Western blotting before. Non perfused liver sections were used to stain ATP8A1 and ATP9A. The slides were incubated in phosphate-buffer saline (PBS) (140 mM NaCl, 2.7 mM KCl, 1.5 mM KH_2_PO_4_, and 8.1 mM Na_2_HPO_4_) at RT in a humid chamber for 2h, and later blocked (2% (w/v) BSA + 0.2% (w/v) Triton X-100) for 1h at RT. Primary antibodies ATP8A1 (1:50 dilution) and ATP9A (1:50 dilution) were exposed to the sections at 4°C overnight. Next morning, the slides were washed twice for 10 min with PBS and stained with secondary antibodies (Alexa flour 488 (1:100); and Cy3^TM^-conjugated goat-anti-mouse (1:100); for 30 min at RT in darkness. ATP8A1 was co-localized with an anti-aminopeptidase-N (APN) monoclonal antibody [19, 20] and ATP9A was co-localized with an anti BSEP polyclonal antibody (rBSEP) [1, 21]. ATP11A (1:50 dilution) and ATP11C (1:50 dilution) immunostaining was performed using a similar protocol but on perfused liver sections. ATP11A was co-localized either using a monoclonal anti RAB7 antibody as late endosomal marker (1:100 dilution) or with a monoclonal anti EEA1 antibody (1:50) as an early endosomal marker, and ATP11C was co-localized with the monoclonal ascites 1/18 as a basolateral marker (19). All slides were mounted using Vectashield Hardset^TM^ with DAPI (Vector Laboratories, Burlingame, CA, USA) and visualized with confocal laser scanning microscopy (Leica DMI6000B, CLSM-Model SP8, Leica, Wetzlar, Germany).

## Results

### Characterization of the rat canalicular membrane proteome

In order to reduce the potential presence of cytosolic and loosely adhering non-transmembrane proteins, cLPM vesicles were extracted in preliminary experiments by alkaline extraction at pH 12.2, alkaline extraction with 1 M NaSCN pH 8.0 or with 100 mM Na_2_CO_3_ and assessed by SDS-PAGE in conjunction with silver staining and Western blotting against rBSEP and rMRP2 [22]. Based on the results (data not shown), extraction of cLPM with Na_2_CO_3_ [23] was chosen as standard pretreatment. Extracted cLPM vesicles were thoroughly digested by PNGase F for removal of N-glycans, followed by sequential enzymatic digestion with trypsin and chymotrypsin / trypsin. SCX chromatography and high mass accuracy mass spectrometry was used to achieve a comprehensive coverage of the membrane proteins. We obtained a total of 25742 peptides corresponding to 2449 proteins from 3 individual biological replicates as shown in S1A Fig and S1B Fig and listed in S2 Table with a false discovery rate of 1% at peptide and at protein level. It should be noted that Rep_3 represents a replicate of the same biological sample measured on two different LC-MS systems (see Material and Methods) and hence has a higher peptide and protein count than the other biological replicates. The two determinations had an overlap of ~63% at the protein level (data not shown).

**Fig 1:**
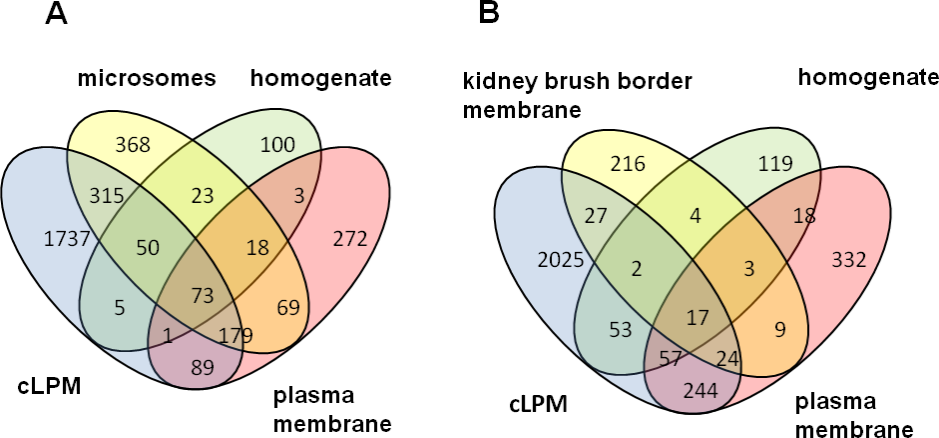
Comparison of rat cLPM proteomics with previous rat liver and brush border membrane proteomic studies. A: Representation of unique and overlapping proteins between rat cLPM compared with previous proteomics studies conducted on rat liver: cLPM this study, total [24], microsomes [25], plasma membrane [26] B: Representation of unique and overlapping proteins between rat cLPM and brush broader membrane proteome: cLPMthis study, kidney brush border membrane [27], total [24], plasma membrane[26].

We first compared our dataset with previously published rat liver and kidney brush border membrane proteome studies [24–27] as shown in Fig 1A and 1B. Even though there are numerous proteomics studies conducted on rat liver [24–26, 28], none has neither specifically targeted membrane proteins nor analyzed highly purified cLPM. This can explain the absence of a relatively large subset (~49%, 1723 proteins) of our proteins from previous studies and hence the identification of additional proteins in the current study as shown in Fig 1A. A comparison of the liver proteome with the kidney brush border proteome (Fig 1B) gave only around 70 common proteins. Additionally to check the transmembrane helices (TM) in our list of proteins, we used the transmembrane topology prediction Phobius embedded in the UniProt knowledge database (http://www.uniprot.org/) and the transmembrane hidden Markov model (TMHMM server; http://www.cbs.dtu.dk/services/TMHMM/) algorithm 33 (S3 Table). This helped to predict the theoretical TM of the identified proteins (2449) from our study. This prediction tool represented transmembrane helices in more than 39% (955) of the identified proteins as shown in S3 Table. This supports the efficiency of the protocol used for eliminating cytoplasmic and loosely adhering proteins.

Manual sorting of the data using MS-Excel pivot tables resulted in identification of 112 kinases, 39 phosphatases, 91 transporters, and 47 ATPases. Among the identified transporters we found 39 members of SLC families and 10 members of the ABC transporter family and in addition members of P4- (ATP8A1, ATP8B1, ATP9A, ATP11A, ATP11C) and P5- (ATP13A1) ATPases (S2 and S4 Tables). Most of the identified P4- and P5-ATPases were not previously known to be expressed at the protein level in rat liver.

### Validation of P4-ATPases expressions by qRT-PCR and Western blot analysis

**Fig 2:**
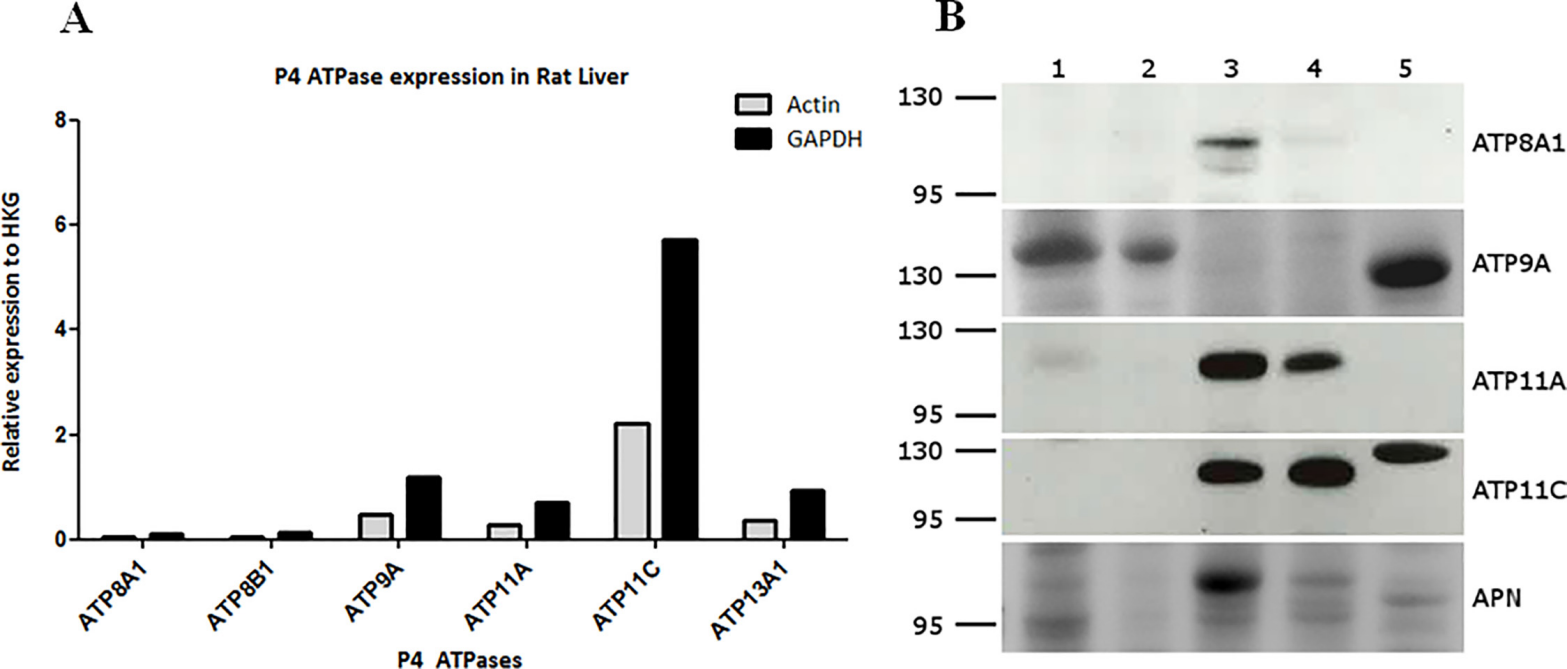
RT-PCR and Western blot analysis on P4- and P5-ATPases. (**A**) Representation of relative mRNA expression in rat liver of the P4- and P5-ATPases by semi quantitative RT-PCR analysis. The expression is given is relative to the house keeping genes actin and GAPDH as described in Materials and Methods. Data are the mean of two independent determinations (biological replicates) with 3 technical replicates each. (**B**) Western blot analysis of P4-ATPases in different liver subcellular fractions. The lanes were loaded with 1. homogenate 2. microsomes, 3. cLPM 4. bLPM, 5.mitochondria. For detecting the P4-ATPases and the canalicular marker APN, the following amount of protein was loaded for each lane: ATP8B1: 100 μg; ATP9A: 150 μg; ATP11A: 100 μg, ATP11C: 50 μg; APN: 150 μg

For validation purposes and for comparing expression levels, we analyzed the mRNA and protein levels using qRT-PCR and Western blotting with commercially available primers (S1 Table) and antibodies, respectively. qRT-PCR showed highest expression of ATP11C compared to the other P4- and P5-ATPases (Fig 2A), confirming the findings from previous studies showing a wide tissue distribution of various members of mammalian P4-ATPases [29]. In Western blot analysis ATP8A1 is observed to be highly expressed in the cLPM (Fig 2B, lane 3). However, other members of P4-ATPase (ATP9A & ATP11A) showed a broader expression in various subcellular liver fractions. ATP11C shows high expression in the bLPM membrane fraction as shown in Fig 2B, lane 4. In addition, we used APN as an established canalicular marker displaying a strong staining in the cLPM (Fig 2B, lane 3), which is virtually absent in the homogenate (Fig 2B, lane 1). This demonstrates the high enrichment of canalicular membranes [10] in our cLPM fraction. As P4-ATPases are involved in lipid homeostasis, we concentrated our following work on the P4-ATPases.

### Immunohistochemical localization of P4-ATPases in rat hepatocytes

The present knowledge on the cellular function of P4-ATPases is mostly derived from studies conducted in non-mammalian species such as *Saccharomyces cerevisiae*, *Arabidopsis thaliana*, *Caenorhabditis elegans*, where they play a crucial role in the biogenesis of intracellular transport vesicles, polarized protein transport and protein maturation [30]. We therefore performed immunohistochemical experiments to test for the expression of class 2 P4-ATPase members.

#### ATP8A1

**Fig 3:**
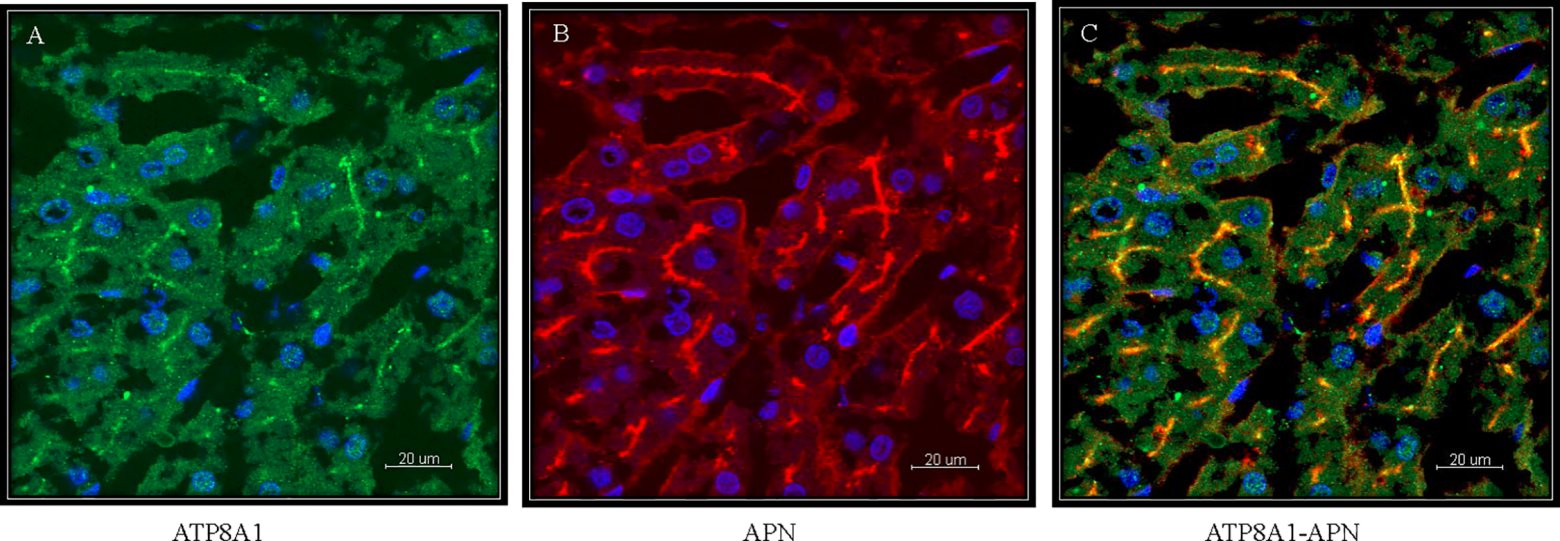
Immunohistochemical localization of ATP8A1 in non-perfused rat liver 5μm cryosections. (**A**) ATP8A1. (**B**) Aminopeptidase-N localized on the canalicular membrane (**C**) Colocalization of ATP8A1 and aminopeptidase-N illustrates the expression of ATP8A1 at canalicular membrane of rat hepatocytes. Scale bar: 20 μm

Non-perfused 5μm liver cryosections were incubated with ATP8A1 antibody and immunoreactivity was observed at the cLPM of rat hepatocytes (Fig 3A). To confirm the canalicular expression of ATP8A1, we co-localized it with the cLPM marker APN [20]. The co-localization is shown in Fig 3C and confirms ATP8A1 expression in the cLPM of hepatocytes, which is in line with the Western blot analysis (Fig 2B, lane 3).

#### ATP9A

**Fig 4:**
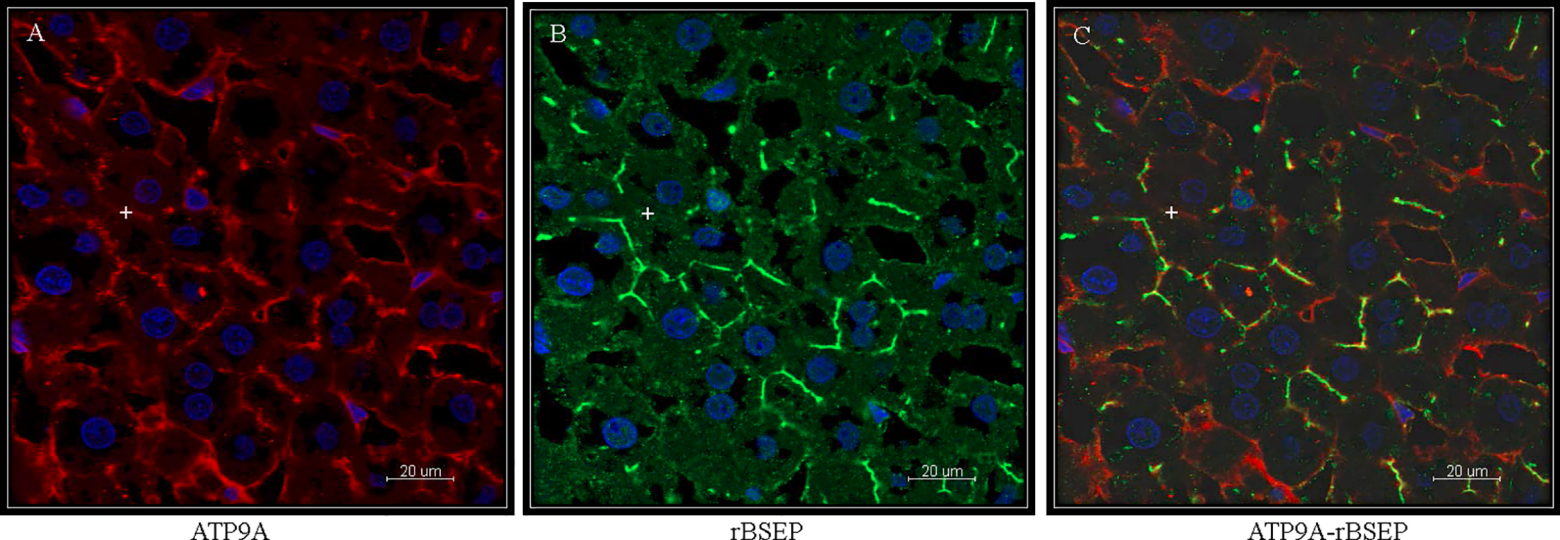
Immunohistochemical localization of ATP9A in non-perfused rat liver 5μm cryosections. (**A**) ATP9A. (**B**) BSEP expressed at the canalicular membrane of rat hepatocytes. (**C**) Colocalization of ATP9A and BSEP shows ATP9A expression at the canalicular membrane of rat hepatocytes. Scale bar: 20 μm

ATP9A was localized with a monoclonal antibody in non-perfused 5μm liver cryosections. The expression was detected at the cLPM of the hepatocytes as shown in Fig 4A colocalizing with rBSEP [21]. It has however to be pointed out that this colocalization was not complete and we also observed some staining at the bLPM. Taken the immunohistochemical findings together with the results from the Western blotting (Fig 2B) with a detection of the protein in microsomes and in a fraction enriched in mitochondria, the localization of ATP9A is not domain-specific but at the entire hepatocyte plasma membrane in rat liver as well as in intracellular membranes.

#### ATP11A

**Fig 5:**
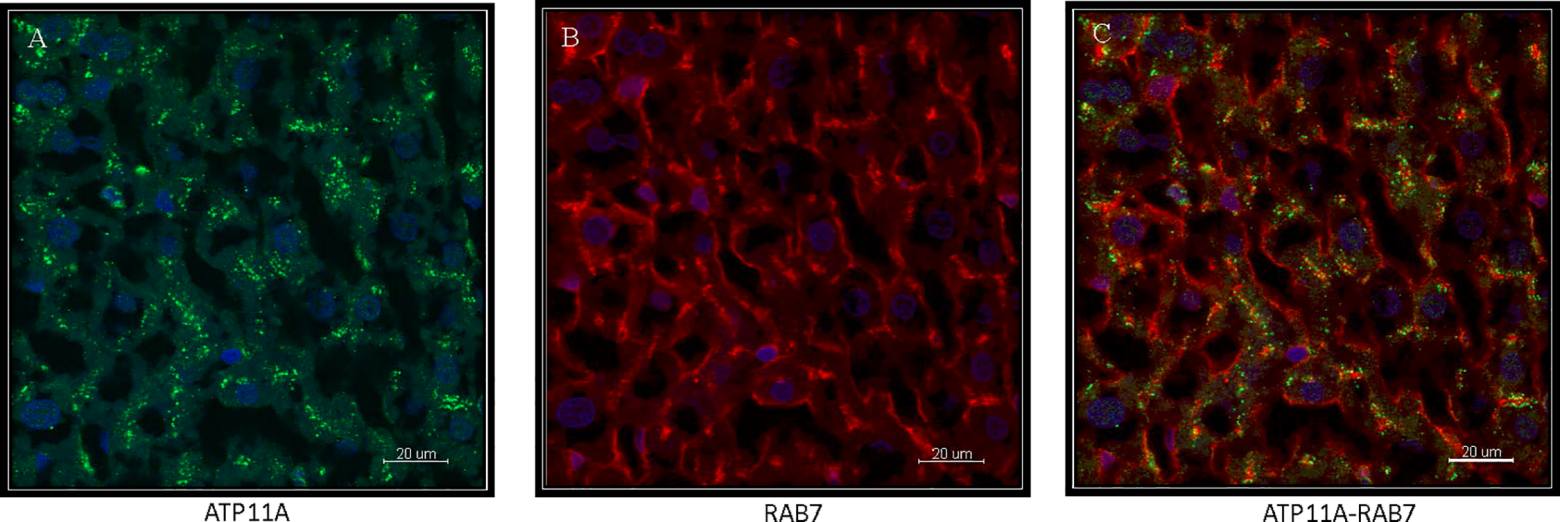
Immunohistochemical localization of ATP11A in perfused rat liver 5μm cryosections. (**A**) ATP11A. (**B**) Expression of RAB7, a late endosome marker. (**C**) Colocalization of ATP11A and RAB7 shows considerable overlap demonstrating ATP11A in a late endosomal compartment. Scale bar: 20 μm

**Fig 6:**
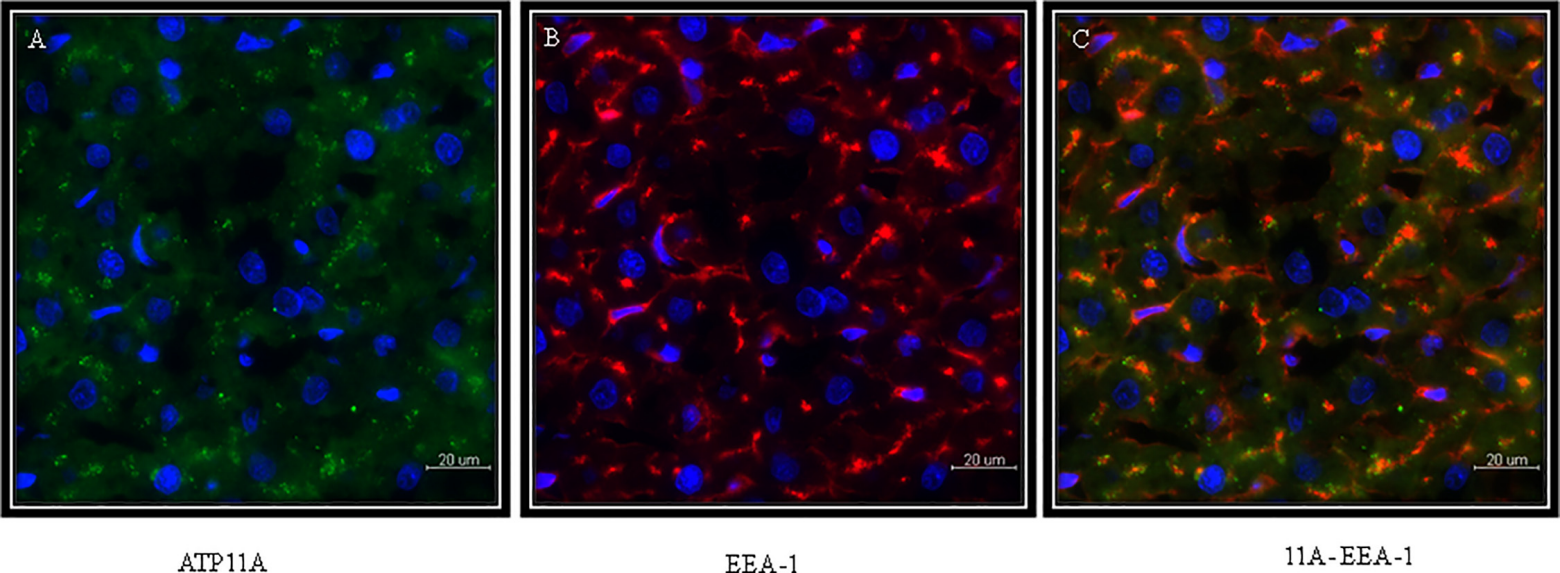
Immunohistochemical localization of ATP11A in perfused rat liver 5μm cryosections. (**A**) ATP11A. (**B**) Expression of EEA1, an early endosome marker. (**C**) Colocalization of ATP11A and EEA1 shows partial overlap demonstrating ATP11A in an early endosomal compartment. Scale bar: 20 μm

ATP11A was studied in perfused rat liver 5μm liver cryosections shown in Fig 5A. ATP11A localized to intracellular vesicular structures, reminiscent of an endosomal compartment. Therefore colocalization analysis of ATP11A was performed with a monoclonal antibody against RAB7 expressed in late endosomes. Fig 5C shows a considerable overlap in the staining of the two proteins making it likely that ATP11A is expressed in the late endosomal compartment of hepatocytes. To obtain more insights into the expression of ATP11A in the endosomal compartment, we also co-localized this P4-ATPase with the early endosomal marker EEA1 (Fig 6B) [31]. Again, we did not find a complete overlap (Fig 6C). This suggests that ATP11A is expressed throughout the endosomal compartment.

#### ATP11C

For ATP11C localization, perfused 5μm liver cryosections were used. The immunostaining was variable with sometimes a weak labelling of the bLPM and a consistent intracellular punctate staining (data not shown) Using the monoclonal antibody 1/18 as a marker for the bLPM [20], we found a very weak colocalization between ATP11C and 1/18 (data not shown). The data from the Western blot in Fig 2B clearly demonstrate expression of ATP11C in the bLPM fraction. This result is also supported by a recent report demonstrating the expression of ATP11C at the bLPM of mouse hepatocytes [32].

## Discussion

In this study, we performed a proteomic analysis of highly purified rat cLPM, which were specifically enriched for membrane and membrane associated proteins. The two main findings of this study are: First, about half of the proteins identified in the present study are found for the first time in rat liver at the protein level [24–26]. Second, we found several P4-ATPases to be expressed in hepatocytes, suggesting that membrane lipid homeostasis may be maintained by a complex array of energy-requiring ATP-dependent systems like P4-ATPases.

As we anticipated that membrane proteins might be more difficult to detect in the mass-spectrometric analysis chosen, we tried to enrich this class of proteins as much as possible in cLPM. This is the most likely explanation that about 49% of the identified proteins have not been described in previous analyses of liver proteomes. The analysis of the identified proteins using the TMHMM server strongly supports the success of our carbonate extraction method. TMHMM showed transmembrane (TM) helices in 955 proteins. TMHMM is one of several computational tools enabling to predict the transmembrane helices of proteins, but it is important to note that different informatics tools for TM prediction may produce different results related to the number and/or location of TM regions [33]. Our main interest for this study was in the identification of transport proteins. Therefore, we were in the present study only interested to predict the transmembrane helices for assigning membrane association and hence did not perform additional analyses. However, it can be assumed that the proportion of membrane associated proteins would be higher if fatty acid and glycolipid anchored proteins would be included as well.

Analyzing functional properties of the proteins in our list, we identified transporters with obvious roles in canalicular bile formation (e.g. MRP2, BSEP, MDR3, ABCG5/ABCG8; S2 and S4 Tables), along with members of P4-ATPases (ATP8B1), which have an important role in phospholipid translocation across membranes. In addition to well established canalicular proteins, we also identified proteins expressed in different membrane domains or organelles, e.g. at the bLPM (Na+K-ATPase, MRP6; S2 and S4 Tables) or at the endoplasmic reticulum (sarcoplasmic/endoplasmic reticulum calcium ATPase 2 (SERCA 2), UDP-glucuronosyltransferase 2B2) as well as SLC-transporters known to be expressed in membranes other than the cLPM. That is not surprising for two reasons: First, even though the method used to isolate cLPM yields a very pure canalicular membrane fraction, it still contains some contaminating fractions from the bLPM as well as from intracellular organelles, for example about 9% microsomal membranes [10, 11]. Second, the analytical method chosen (electrospray ionization) identifies peptides, which are easily ionized and can produce MS/MS spectra leading also to the detection of low abundant proteins (which can be expected for a contaminating membrane fraction). In the further analysis we focused on selected proteins of interest, namely P4-ATPases, with respect to their expression and to their subcellular localization [34].

Members of the P4-ATPases have been implicated in the flipping of phospholipid across membranes [29, 30, 35, 36] from the exoplasmic to the cytoplasmic leaflet. The first evidence showing the involvement of P4-ATPases in phospholipid translocation comes from a study conducted on the P4-ATPase Drs2p expressed in *S*. *cerevisiae*. That protein was demonstrated to be involved in the transport of fluorescently (NBD)-labeled analogs of phosphatidylserine (PS) and phosphatidylethanolamine across the plasma membrane and across the Golgi membranes of yeast [29]. However, the physiological roles and the cellular functions and localizations of many of the P4-ATPases are still largely unexplored in mammalian tissues. Mutations in the gene coding for ATP8B1 are known to lead to severe liver disease in humans, namely progressive familial intrahepatic cholestasis 1 (PFIC1), and benign recurrent intrahepatic cholestasis type 1 (BRIC1) [8]. The inactivation of the gene coding for ATP11C in mice leads to hyperbilirubinemia and hypercholanemia in the absence of elevated transaminases [37].

We first verified the expression of the investigated P4- and P5-ATPases at the mRNA level. Of these five ATPases, ATP11C gave the highest expression at the mRNA level, but, interestingly, was not detected at the protein level in a liver homogenate (Fig 2B). Not all of these ATPases were found to be specifically expressed in the cLPM, but showed a broader organelle distribution suggesting that multiple different hepatocyte membranes may require phospholipid translocators for maintaining lipid homeostasis. For example, ATP8A1 was only found in cLPM and ATP11C predominantly in bLPM, suggesting a polar expression at the hepatocyte plasma membrane

ATP8A1, which belongs to the class 1a of the P4-ATPases, is expressed at the canalicular membrane. Of note, the bovine homologue of ATP8A1 was first isolated from bovine and mouse chromaffin granules of adrenal glands [38]. Hence, the subcellular localization of ATP8A1 seems to be tissue specific. Later it was found in erythrocyte precursors and at the membranes of mature erythrocytes. Functionally, ATP8A1 was associated with inward PS flipping activity [39]. In mice with an inactivated *Atp8a1* gene, erythrocytes displayed no PS externalization, which could be due to an overexpression of ATP8A2 [40]. Furthermore, in Chinese hamster ovary cells ATP8A1 was involved in cell motility [41]. Taking all this information together it is likely that ATP8A1 may display ATP-dependent phospholipid translocase activity at the cLPM, but its exact substrate spectrum remains uncharacterized so far.

ATP9A belongs to class 2 of the P4-ATPase family together with ATP9B in mammals [30]. ATP9A is to date relatively unexplored, but protein localization to endosomes and trans-Golgi network in HeLa cells was reported after transfection [42]. In INS-1 832/13 cells, it was found to be expressed at the plasma membrane and in the trans-Golgi network and in insulin containing granules [43]. This study found that a knockdown of ATP9A leads to an inhibition of glucose-induced insulin secretion form INS-1 832/13 cells. In rat hepatocytes ATP9A is clearly expressed in the cLPM (Fig 4A). Western blot analysis of different subcellular liver fractions suggests a broader subcellular expression in hepatocytes (Fig 2B), which is in line with the above mentioned findings.

ATP11C belongs to class 6 of P4-ATPases and is preferentially involved in the flipping of PS and phosphatidylethanolamine but not of PC across plasma membranes [44]. In our study the investigation of the expression of ATP11C was challenging and yielded variable results. ATP11C was observed at the bLPM as well as in intracellular vesicles, but not in the cLPM. This finding is remarkable given that mice with inactivated *Atp11c* display an elevation of cholephilic compounds (bilirubin and bile salts) in serum without an elevation of transaminases. Indeed, it has recently been demonstrated that in mice ATP11C is expressed at the bLPM [32] and that mice with an inactivated *Atp11c* gene have lower protein expression levels of basolateral organic anion transporters such as the sodium-taurocholate cotransporting polypeptide or the organic anion transporting polypeptides OATP1A4 and OATP1B2, which are involved in the transport of bilirubin and bile salts [32, 45, 46]. The positive signal in cLPM on Western blots (Fig 2B) might be due to the presence of intracellular vesicles in this membrane fraction. A role of ATP11C in maintaining biophysical properties (e.g. lipid asymmetry) of cell membranes is supported by the observation that *Atp11c* knock-out mice suffer from lymphopenia [37], which may be due to a defect in the transition from the pro- to the pre-B cell stage of B-cells, a process requiring clathrin-mediated endocytosis of ligand-bound interleukin-7 receptor.

ATP11A, which is a member of class 6 of P4-ATPases family is a possible negative predictive marker for metachronous metastasis in colorectal cancer patients [47] and may confer resistance to cancer treatment with farnesyltransferase inhibitors [48]. In addition, in a genome-wide association study, a polymorphism in the 3’-UTR of ATP11A was found to be a susceptibility locus for pulmonary fibrosis [49]. These findings demonstrate that ATP11A may be involved in a wide variety of different cellular processes.

Several P4-ATPases require the association with CDC50 proteins for their exit from the endoplasmic reticulum [30]. We also identified CDC50A (TMEM30a) in our proteome (S2 and S4 Tables). The biosynthesis of phospholipids at the endoplasmic reticulum occurs at the cytoplasmic side and requires a phosphatidylcholine flippase [50]. Evidence for an ATP-independent phosphatidylcholine flippase activity has also been found in rat cLPM vesicles [51]. In this respect, it is interesting to note that we identified the scramblase anoctamine in our proteome (S2 Table). The exact subcellular localization of this protein as well as its role as an equilibrating lipid translocator in hepatocytes remains to be worked out.

The comparison of the canalicular proteome with the proteome of rat kidney brush border membrane (Fig 1B) shows a surprisingly low overlap. One reason for this may be methodological differences, while an alternate reason could be that the kidney brush border membrane is not facing such a hostile environment as the cLPM in liver.

In conclusion, the determination of the rat canalicular proteome revealed the presence of two new P4-ATPases in this membrane. While the exact function of these ATPases is not known, it is tempting to speculate that these two enzymes may contribute to lipid homeostasis and hence contribute to the remarkable stability of the cLPM against the attacks of a highly concentrated detergent solution.

## Supporting Information

S1 Fig Comparison of peptides and proteins of cLPM from three biological replicates. A. peptides, B. proteins. Rep_3 was made by two replicates of one biological sample determined as described in Materials and Methods.
(TIF)

S1 Table Primers used for mRNA expression studies (XLSX)

S2 Table List of peptides and proteins of cLPM (XLSX)

S3 Table TMHMM analysis of cLPM proteins. The information was retrieved from the UniProt Knowledgebase (http://www.uniprot.org (XLSX)

S4 Table Summary of proteins specifically discussed in this study. For all proteins, both the generic names as well as the UniProt entries are given.
(XLSX)

## Abbreviations

PC: phosphatidylcholine
BSEP: bile salt export pump
cLPM: canalicular liver plasma membrane
bLPM: basolateral liver plasma membrane
SCX: strong cation exchange
MS: mass spectrometry
IHC: immunohistochemistry
qRT-PCR: real time polymerase chain reaction
TM: transmembrane
