# Supplementary figures and images for: Proteomic Analysis of the Rat Canalicular Membrane Reveals Expression of a Complex System of P4-ATPases in Liver
Source: PLoS One. 2016 Jun 27;11(6):e0158033. doi: 10.1371/journal.pone.0158033 (PMC4922570; doi:10.1371/journal.pone.0158033)

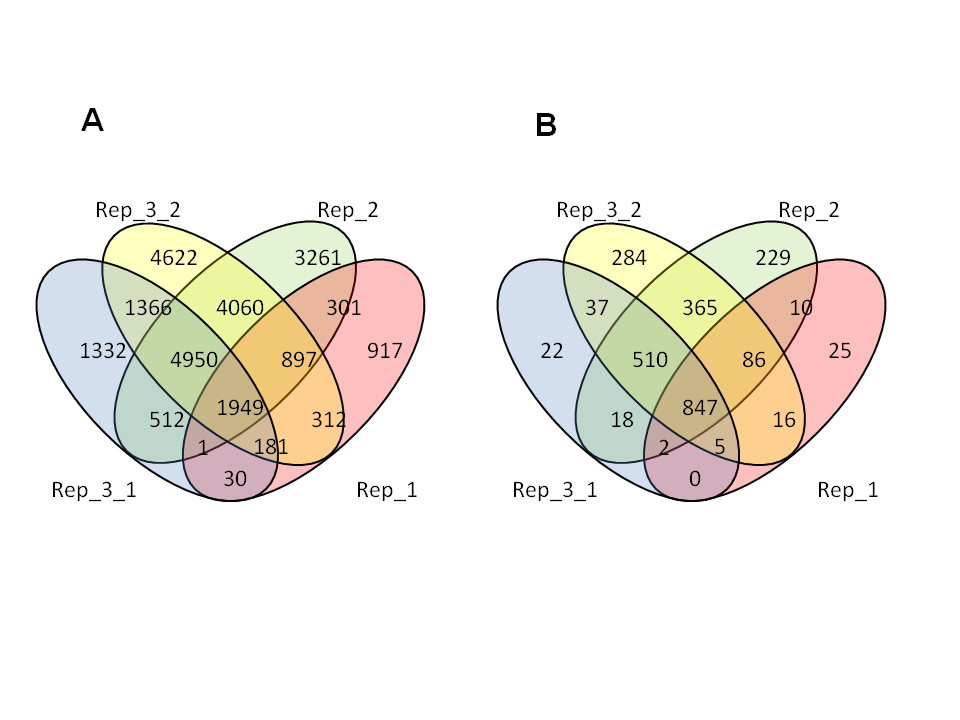

Supplement: S1 Fig — A. peptides, B. proteins. Rep_3 was made by two replicates of one biological sample determined as described in Materials and Methods. (TIF) [file pone.0158033.s001.tif]
